# Intrathoracic lipoma of chest wall misdiagnosed as liposarcoma with malignant features based on radiological image and intraoperative findings

**DOI:** 10.1093/jscr/rjad046

**Published:** 2023-02-10

**Authors:** Sachie Koike, Masahisa Miyazawa, Nobutaka Kobayashi, Hidetoshi Satomi

**Affiliations:** Department of Thoracic Surgery, Japanese Red Cross Society Nagano Hospital, Nagano, Nagano, Japan; Division of General Thoracic Surgery, Department of Surgery, Shinshu University School of Medicine, Matsumoto, Nagano, Japan; Department of Thoracic Surgery, Japanese Red Cross Society Nagano Hospital, Nagano, Nagano, Japan; Department of Thoracic Surgery, Japanese Red Cross Society Nagano Hospital, Nagano, Nagano, Japan; Department of Pathology, Japanese Red Cross Society Nagano Hospital, Nagano, Nagano, Japan

## Abstract

Lipomas are common benign tumors, which are usually located in the subcutaneous tissue. It is relatively rare for lipoma to occur in the intrathoracic cavity, and it is clinically difficult to distinguish it from liposarcoma. We present the case of a 72-year-old man with a chest wall tumor preoperatively diagnosed as liposarcoma, with tumor enlargement with radiological image change to heterogenous and 18F-fluorodeoxyglucose positron emission tomography uptake. The tumor was resected along the chest wall, lung and diaphragm because of dense adhesions. The tumor was diagnosed as lipoma with fat necrosis and inflammatory changes.

## INTRODUCTION

Lipomas of the intrathoracic cavity are relatively rare and are reported to be located in the intracardiac, pleural and intramuscular regions. There are few reports of chest wall lipomas, and it is always clinically difficult to distinguish them from liposarcomas [1]. Herein, we report a case of lipoma of the chest wall preoperatively diagnosed as liposarcoma due to malignant feature based on radiological image and intraoperative findings.

## CASE REPORT

**Figure 1:**
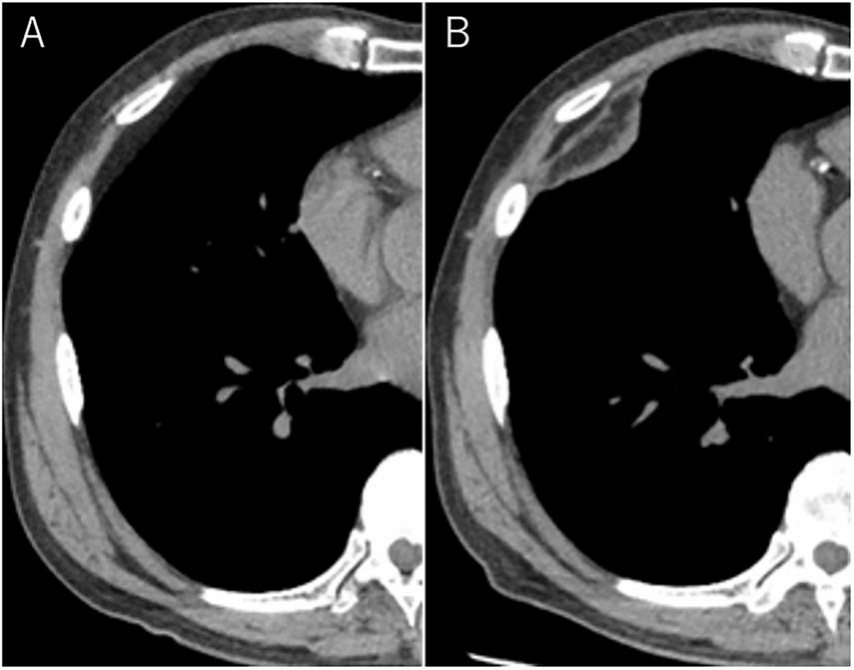
Mixed density mass located in front of thoracic wall; the size was 8.0 × 5.0 × 3.0 cm (**B**); (**A**) The tumor seen as thoracic wall fat (7.8 × 4.8 × 1.2 cm) (arrow) on CT 1 year ago; (B) the size increased and the density has changed to heterogenous in 1 year.

**Figure 2:**
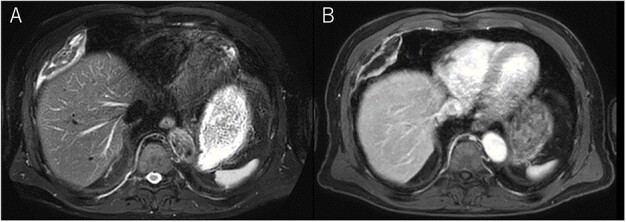
Chest MRI; (**A**) T2-weighted imaging showed several high signal foci in the tumor; (**B**) irregular marginal enhancement was observed.

**Figure 3:**
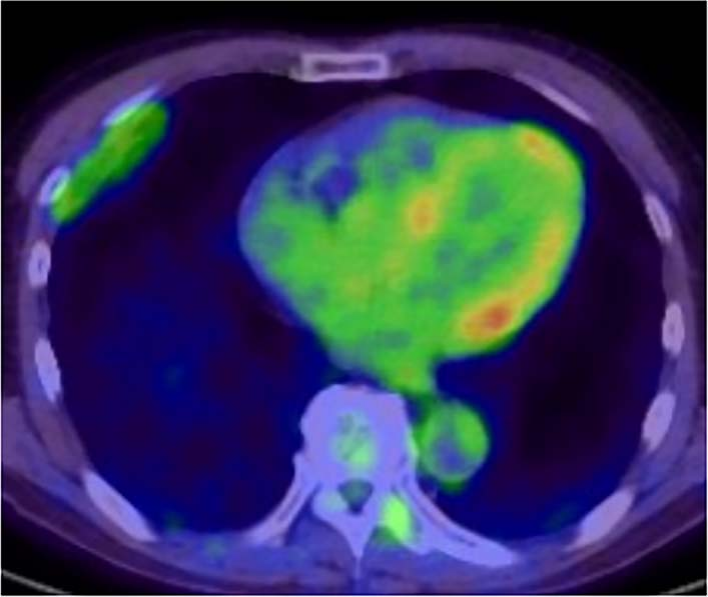
18F-FDG-PET uptake by the tumor; maximum standard uptake value was 3.78.

**Figure 4:**
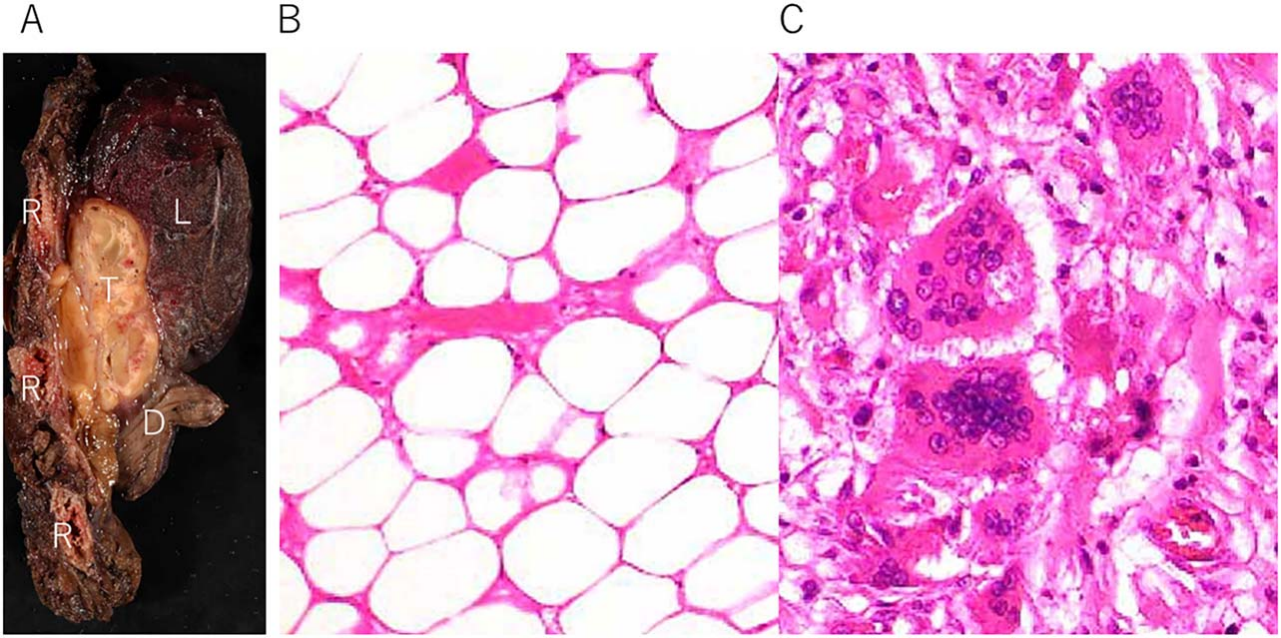
(**A**) Tumor (T) with fibrous adhesion between ribs (R), lung (L) and diaphragm (D); (**B**) matured fat tissue consist the tumor; (**C**) marginal area of the tumor fat necrosis and inflammatory changes with foamy macrophage and multinucleated giant cells.

A 72-year-old man with a history of hypertension and left renal cancer (Stage I) was referred to us with a mass in the right chest wall on post-operative follow-up chest computed tomography (CT) for renal cancer. He had a smoking history of 20 pack-years and had no exposure to environmental fumes or dust. Physical examination results were unremarkable. The laboratory findings were within normal limits. Pulmonary function tests and cardiovascular examinations revealed normal results. Chest CT revealed mixed density mass (8.0 × 5.0 × 3.0 cm) located in front of thoracic wall in the third to sixth right intercostal space. The tumor can be revealed as thoracic wall fat (7.8 × 4.8 × 1.2 cm) on CT 1 year before (not considered as abnormal), and it was progressively increased in size and the density changed (Fig. 1A and B). Magnetic resonance imaging (MRI) showed a fatty mass of heterogenic density. T2 high foci (Fig. 2A) and irregular marginal enhancement of the tumor were observed (Fig. 2B). Maximal standard uptake value (SUVmax) of 18F-fluorodeoxyglucose positron emission tomography (FDG-PET) was 3.78 (Fig. 3). Based on these radiological image findings, we scheduled surgery with suspicion of liposarcoma. During the surgery, the patient was placed in the lateral decubitus position. We made 1.5-cm incision in the sixth intercostal space along the posterior axial line for thoracoscopy. We found dense adhesions between the chest wall tumor, lung (front part of all three lobes of the right lung) and diaphragm. We made a 30-cm incision in the fourth intercostal space and resected the tumor along with lung (wedge resection of the front part of all three lobes of the right lung), diaphragm and third to sixth ribs and intercostal muscle. The chest wall defect was 25 × 15 cm and the diaphragm defect was 8 × 5 cm. For reconstruction, the mesh was placed and sutured to the diaphragm and the chest wall. Pathological examination revealed the well-circumscribed tumor with fibrous adhesion between the ribs, lung and diaphragm (Fig. 4A). Microscopically, the tumor consisted of mature fat tissue. There were fat necrosis inflammatory changes in the marginal area of the tumor with foamy macrophages and multinucleated giant cells (Fig. 4B and C). Fluorescence *in situ* hybridization examination for murine double-minute 2 was negative. Based on these findings, a chest wall lipoma was diagnosed. The post-operative course was uneventful. The patient was followed up for 24 months without evidence of recurrence.

## DISCUSSION

Lipomas are common benign tumors, and during treatment of deep-seated lipoma, differential diagnosis between well-differentiated liposarcomas should be considered [1] . Lipomas can be treated successfully with local or marginal excision, whereas well-differentiated liposarcomas need to be treated with wide local excision because of the high rate of local recurrence (10–52%) [2]. In addition, well-differentiated liposarcomas require 5–10 years of follow-up with delayed dedifferentiation at that timing [3, 4]. Due to these differences in treatment and prognosis, it is important to preoperatively distinguish between these lesions.

Several critical radiological image findings have been reported for the differential diagnosis. Lipomas are discrete, encapsulated, homogeneous fatty masses on CT and MRI. On the other hand, thickened or nodular septa (>2 mm), prominent foci of high T2 signal and prominent areas of enhancement on MRI have been reported to be features suspicious of liposarcoma [5, 6]. In addition, tumor size >10 cm and fat content <75% have been reported as radiological features of liposarcoma [6]. SUVmax of FDG-PET reported that lipomas measured 0.8 ± 0.2 and well-differentiated liposarcomas measured 2.3 ± 1.2 [7].

In the present case, tumor enlargement with heterogenic change of the density, thickened septa >2 mm, prominent foci of high T2 signal and prominent areas of enhancement on MRI were seen on preoperative CT and MRI. In addition, the SUVmax of FDG-PET was higher than that of simple lipoma (SUVmax = 3.78). These findings led to a misdiagnosis for liposarcoma. Furthermore, intraoperative findings of dense adhesion between the tumor and surrounding structures strengthen our suspicion of liposarcoma. We could not find another case of intrathoracic lipoma of the chest wall with density change on radiological image and dense adhesion between the surrounding structures.

Pathological findings in this case showed fat necrosis and inflammatory changes in the marginal area of the tumor, with foamy macrophages, and multinucleated giant cells. These changes may cause the malignant featured images, such as heterogenic density, enhancement on MRI and high SUVmax of FDG-PET. In addition, these changes may cause dense adhesion. Several lipomas with fat necrosis has been reported [8], but none of them have intrathoracic origin. In addition, no other cases with conspicuous radiological image change have been reported as in our case.

In conclusion, we encountered a case of chest wall lipoma misdiagnosed as liposarcoma, with malignant features based on radiological image and intraoperative findings, and performed wide local excision. Fat necrosis and inflammation may be the cause of this clinical course.
